# A machine learning model based on ultrasound image features to assess the risk of sentinel lymph node metastasis in breast cancer patients: Applications of scikit-learn and SHAP

**DOI:** 10.3389/fonc.2022.944569

**Published:** 2022-07-25

**Authors:** Gaosen Zhang, Yan Shi, Peipei Yin, Feifei Liu, Yi Fang, Xiang Li, Qingyu Zhang, Zhen Zhang

**Affiliations:** ^1^ Department of Ultrasound, First Affiliated Hospital of China Medical University, Shenyang, China; ^2^ Department of Ultrasound, Binzhou Medical University Hospital, Binzhou, China; ^3^ Department of Ultrasound Medicine, Peking University People’s Hospital, Beijing, China; ^4^ College of Information Science and Engineering, Northeastern University, Shenyang, China

**Keywords:** breast cancer, ultrasound signs, sentinel lymph node metastasis, XGBoost, SHAP

## Abstract

**Background:**

This study aimed to determine an optimal machine learning (ML) model for evaluating the preoperative diagnostic value of ultrasound signs of breast cancer lesions for sentinel lymph node (SLN) status.

**Method:**

This study retrospectively analyzed the ultrasound images and postoperative pathological findings of lesions in 952 breast cancer patients. Firstly, the univariate analysis of the relationship between the ultrasonographic features of breast cancer morphological features and SLN metastasis. Then, based on the ultrasound signs of breast cancer lesions, we screened ten ML models: support vector machine (SVM), extreme gradient boosting (XGBoost), random forest (RF), linear discriminant analysis (LDA), logistic regression (LR), naive bayesian model (NB), k-nearest neighbors (KNN), multilayer perceptron (MLP), long short-term memory (LSTM), and convolutional neural network (CNN). The diagnostic performance of the model was evaluated using the area under the receiver operating characteristic (ROC) curve (AUC), Kappa value, accuracy, F1-score, sensitivity, and specificity. Then we constructed a clinical prediction model which was based on the ML algorithm with the best diagnostic performance. Finally, we used SHapley Additive exPlanation (SHAP) to visualize and analyze the diagnostic process of the ML model.

**Results:**

Of 952 patients with breast cancer, 394 (41.4%) had SLN metastasis, and 558 (58.6%) had no metastasis. Univariate analysis found that the shape, orientation, margin, posterior features, calculations, architectural distortion, duct changes and suspicious lymph node of breast cancer lesions in ultrasound signs were associated with SLN metastasis. Among the 10 ML algorithms, XGBoost had the best comprehensive diagnostic performance for SLN metastasis, with Average-AUC of 0.952, Average-Kappa of 0.763, and Average-Accuracy of 0.891. The AUC of the XGBoost model in the validation cohort was 0.916, the accuracy was 0.846, the sensitivity was 0.870, the specificity was 0.862, and the F1-score was 0.826. The diagnostic performance of the XGBoost model was significantly higher than that of experienced radiologists in some cases (P<0.001). Using SHAP to visualize the interpretation of the ML model screen, it was found that the ultrasonic detection of suspicious lymph nodes, microcalcifications in the primary tumor, burrs on the edge of the primary tumor, and distortion of the tissue structure around the lesion contributed greatly to the diagnostic performance of the XGBoost model.

**Conclusions:**

The XGBoost model based on the ultrasound signs of the primary breast tumor and its surrounding tissues and lymph nodes has a high diagnostic performance for predicting SLN metastasis. Visual explanation using SHAP made it an effective tool for guiding clinical courses preoperatively.

## Introduction

Breast cancer is the most common malignancy in women, and its incidence is increasing annually (1). Whether sentinel lymph node (SLN) metastases have important clinical significance for breast cancer staging, surgical selection, and prognosis is still being determined. Sentinel lymph node biopsy (SLNB) is the gold standard for diagnosing SLN metastasis of breast cancer. An invasive method, SLNB may cause complications such as infection at the puncture site and hematoma (2). Moreover, SLNB has a false-positive rate of 5%–10% (3), which leads to the possibility of secondary surgery. This urgently requires imaging to accurately determine the status of SLNs, to avoid extensive lymph node dissection and minimize the trauma to patients.

Ultrasonography has become the preferred method for breast diseases due to its advantages of non-invasiveness, high reproducibility, and good patient cooperation (4). Previous studies (5) showed that the morphological characteristics of the primary breast cancer have a certain relationship with the activity (biological behavior) of the tumor, and its morphology will change with the biological behavior such as lymph node metastasis. This suggests that monitoring the morphological features of breast cancer lesions is of great value in assessing SLN status. Conventional ultrasound can provide macroscopic features of lesions, but the weight of these macroscopic features in relation to SLN status is unclear.

Machine learning (ML) algorithms have been applied in the medical field for outcome prediction, diagnosis, and treatment (6). For example, ML model has been used to differentiate between benign breast nodules and breast cancer based on ultrasound images (7). But the logic and complexity of various ML algorithms are different (8), and there may also be differences in clinical application. A study (9) compared the diagnostic performance of different ML algorithms in the diagnosis of benign and malignant thyroid nodules and found that the random forest (RF) model achieved the best area under the receiver operating characteristic (ROC) curve (AUC) (0.924). Due to the complex nonlinear relationship of some ML algorithms, the model results are difficult to interpret, resulting in a “black-box” problem (10), which limits the clinical application of predictive models. Therefore, the interpretability algorithm of ML model results has become a new research focus (11). SHapley Additive exPlanation (SHAP), based on cooperative game theory, has global and local interpretability, interpreting the predicted value of the model as the sum of the contribution values of each input feature, that is, the shapley value. Compared with other explanation methods in previous literature, SHAP can visualize the prediction process of complex ML prediction models. These advantages make it possible to solve the “black-box” problem of complex ML models with SHAP. At present, SHAP has been successfully applied to intraoperative hypoxemia risk prediction (12) and COVID-19 prognosis assessment (13). Therefore, this study aimed to develop a ML model based on the ultrasound signs of breast cancer lesions and surrounding soft tissues and lymph nodes to predict the risk of axillary lymph node metastasis in breast cancer patients. Using SHAP to visually interpret the prediction results of the ML model, so as to guide the clinical formulation of personalized diagnosis and treatment plans. The SHAP also can promote the clinical application of the prediction model.

## Materials and methods

### Patients

As a retrospective study, this study was approved by the Ethics Committee of the First Affiliated Hospital of China Medical University (AF-SOP-07-1.1-07), which waived the requirement for patient informed consent. All patients in this study underwent ultrasonography of breast cancer lesions and ipsilateral axillary lymph nodes in our department. The inclusion criteria were as follows: (1) patients with primary breast cancer who were first discovered and had no history of other malignancies; (2) no axillary mass was found on physical examination; (3) ultrasound examination within 2 weeks before breast cancer surgery or percutaneous biopsy of the lesion; (4) no other adjuvant therapy such as chemotherapy or radiotherapy was performed before ultrasound examination; (5) and the ultrasound image of the lesion was clear and complete. The exclusion criteria were as follows: (1) non-single lesions; and (2) absence of clinical data and pathology. Finally, we screened 902 consecutive female patients with breast cancer from June 2017 to June 2021 as a primary cohort, constructed a predictive model, and performed internal validation, with a mean age of 49.98 ± 9.81 years (range: 24–86 years). Following the same inclusion and exclusion criteria, we screened another 50 female patients with breast cancer from July 2021 to December 2021 as a validation cohort, with a mean age of 49.82 ± 10.99 years (range: 25–74 years). Pathological findings of all patients in the primary cohort and validation cohort were confirmed postoperatively or after percutaneous needle biopsy.

### Ultrasound evaluation

In this study, three types of ultrasonic diagnostic instruments including Hitachi HI VISION Ascendus (Hitachi Medical Corp., Tokyo, Japan), Canon APLIO 500 (Canon Medical Systems Corp., Otawara, Japan), and SuperSonic Aixplorer (SuperSonic Imagine SA, Aix-en-Provence, France) were used for image acquisition, all of which were equipped with superficial high-frequency linear array probes with a frequency of 8–15MHz. Images were stored in the picture archiving and communication system (PACS) workstation for further analysis. Ultrasound signs of breast cancer lesions images in the PACS workstation were evaluated by two experienced radiologists without knowledge of the exact pathological findings. To assess intra- and inter-observer reproducibility, radiologist A assessed all ultrasound signs in the primary cohort and reassessed these after 1 week to test for intra-observer consistency. All ultrasound signs in the primary cohort were also assessed by radiologist B and compared with those from radiologist A to test for interobserver agreement. The evaluation of ultrasound signs mainly included the shape of the original lesion (oval, round, or irregular), orientation (parallel or not parallel), margin (circumscribed, indistinct, angular, microlobulated, or spiculated), echo pattern (hyperechoic, isoechoic, hypoechoic, anechoic, or complex cystic and solid), posterior features (no posterior features, enhancement, or shadowing), calcifications (no calcification, macrocalcification, microcalcification, or rim calcification), architectural distortion, duct changes, hyperechoic halo, and suspicious lymph nodes. Lymph node classification criteria (14) were evaluated, with categories 1–3 considered benign, and categories 4–6 considered metastatic.

### Data preprocessing

Firstly, we performed univariate analysis of all data from the primary and validation cohorts to screen for ultrasound signs associated with SLN metastasis. Then, it was found by statistics that the number of samples in the SLN transfer group with a small number of samples accounted for 41.4% (394/952) of the total number of samples, which was a balanced sample. Therefore, no relevant processing to deal with data imbalance, such as over-sampled or under-sampled, is performed on the dataset. Finally, in order to speed up the training and improve the diagnostic performance of the model, we standardize and normalize the dataset. For details of data preprocessing, see **Supplementary Material 1**.

### Screening and validation of machine learning models

The 902 samples in the primary cohort were randomly divided into ten parts, and 10-fold cross-validation were performed on 10 ML algorithms such as support vector machine (SVM), extreme gradient boosting (XGBoost), RF, linear discriminant analysis (LDA), logistic regression (LR), naive Bayesian model (NB), k-nearest neighbors (KNN), multilayer perceptron (MLP), long short-term memory (LSTM) and convolutional neural network (CNN). The diagnostic performance of all ML algorithms was adjusted by grid search algorithm to optimize the performance of the ML model and avoid overfitting of the model. We comprehensively evaluated the diagnostic performance of 10 algorithms for predicting breast cancer SLN metastasis using the Average-AUC, Average-Kappa and Average-Accuracy derived from the 10-fold cross-validation. Then, the entire primary cohort was used for 10 ML models for training. The diagnostic performance of the ML model was verified through the validation set data, and the diagnostic performance of all models were evaluated using the ROC curve and the detection error trade-off (DET) curve. Then we used the learning curve to verify the fit of the best performing model. Finally, we compared the best performing ML algorithm with experienced radiologist.

### Visualizing machine learning models

**Figure 1:**
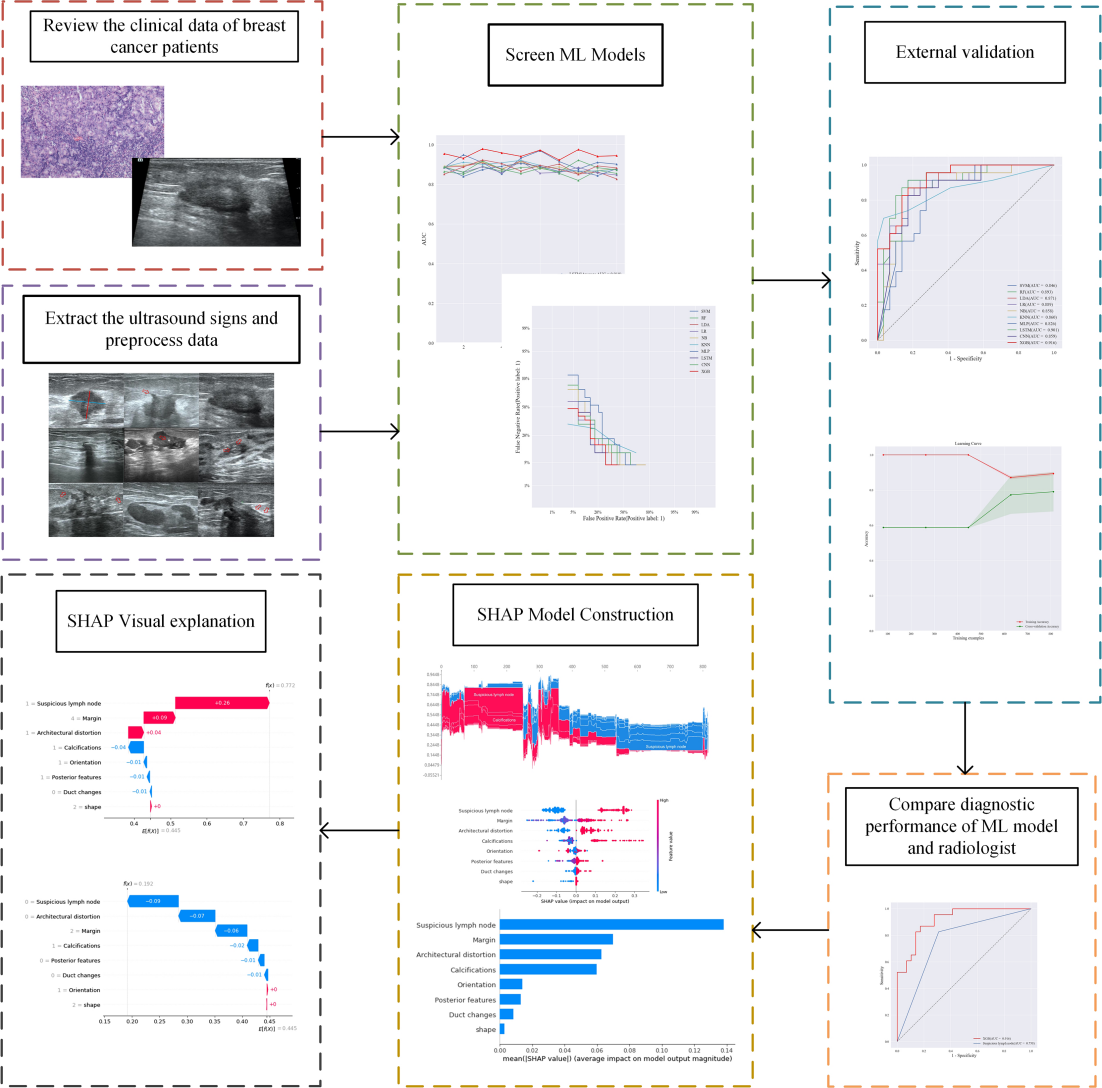
The flowchart of the study.

SHAP measures feature importance by calculating the contribution value, while describing whether the influence of the feature is positive or negative (15). We also utilize SHAP for the visual interpretation of the ML models both holistically and individually (16), which facilitates the clinical applications of the ML model (**Figure 1**).

### Statistical analysis

SPSS (v. 26.0; IBM Corp., Armonk, NY, USA) statistical software was used. Continuous variables were expressed as (*x̄* ± s) using independent samples t-tests and the F-test; categorical variables were expressed as frequencies using the χ^2^ test and Fisher’s exact test. P<0.05 means the difference is statistically significant. Kappa (K) analysis was used to assess intra- and inter-observer agreement. Data analysis used 10 ML algorithms from the Scikit-learn (https://scikit-learn.org/stable/) and Pytorch (https://pytorch.org/) packages in Python (version 3.8). Among them, SVM, XGBoost, RF, LDA, LR, NB, KNN, MLP are from Scikit-learn, and LSTM and CNN are from Pytorch. SHAP (https://github.com/slundberg/shap) was performed using the SHAP Python framework (version 0.40.0).

## Results

### Basic clinical features of breast cancer patients

The pathological results of 902 patients in the primary cohort are shown in **Table 1**, of which 305 were confirmed by surgery, and 597 were confirmed by percutaneous needle biopsy. There were 372 cases in the SLN metastasis group, with an average age of 49.53 ± 9.55 years (range: 26–86 years). There were 530 patients in the SLN non-metastatic group, with an average age of 50.29 ± 9.97 years (range: 24–82 years). There was no significant difference in age between the two groups (t=1.153, P=0.249).

**Table 1:** Pathological types of breast cancer patients in the primary cohort.

Pathology	No. (%) of patients		
	SLN non-metastasis	SLN metastasis	Total
invasive ductal carcinoma	403(76.0%)	356(95.7%)	759(84.2%)
ductal carcinoma in situ	106(20.0%)	14(3.8%)	120(13.3%)
mucinous carcinoma	7(1.3%)	0	7(0.8%)
mixed breast carcinoma	6(1.1%)	1(0.3%)	7(0.8%)
intraductal papillary carcinoma	4(0.8%)	0	4(0.4%)
invasive micropapillary carcinoma	2(0.4%)	1(0.3%)	3(0.3%)
invasive lobular carcinoma	1(0.2%)	0	1(0.1%)
malignant phyllodes tumor	1(0.2%)	0	1(0.1%)
Total	530	372	902

SLN, sentinel lymph node.

### Ultrasound signs of breast cancer lesions

Univariate analysis of ultrasound signs in the primary cohort and validation cohort to screen for risk factors associated with breast cancer SLN metastasis (**Table 2**). We found that the echo pattern (P=0.613) and hyperechoic halo (P=0.855) of lesions were not significantly different between the two groups and were removed. The remaining eight key ultrasound signs, such as shape, orientation, margin, posterior features, calculations, architectural distortion, duct changes and suspicious lymph node, were used for ML algorithms screening.

**Table 2:** Univariate analysis of breast cancer SLN metastasis in primary and validation cohorts.

Variable	Primary cohort (N=902)		*P*-Value	Validation cohort (N=50)		*P*-Value
	SLN metastasis(N=372)	SLN non-metastatic(N=530)		SLN metastasis(N=22)	SLN non-metastatic(N=28)	
shape			<0.001			0.277
oval	0	0		0	0	
round	4 (1.1%)	33 (6.2%)		5 (22.7%)	3 (10.7%)	
Irregular	368 (98.9%)	497 (93.8%)		17 (77.3%)	25 (89.3%)	
orientation			<0.001			0.153
parallel	201 (54.0%)	368 (69.4%)		9 (40.9%)	18 (64.3%)	
not parallel	171 (46.0%)	162 (30.6%)		13 (59.1%)	10 (35.7%)	
margin			<0.001			0.014
circumscribed	5 (1.3%)	20 (3.8%)		0	0	
indistinct	24 (6.5%)	117 (22.1%)		0	4 (14.3%)	
angular	56 (15.1%)	266 (50.2%)		2 (9.1%)	6 (21.4%)	
micro lobulated	75 (20.2%)	93 (17.5%)		4 (18.2%)	10 (35.7%)	
spiculated	212 (56.9%)	34 (6.4%)		16 (72.7%)	8 (28.6%)	
echo pattern			0.613			0.262
anechoic	0	0		0	0	
hyperechoic	0	0		0	0	
isoechoic	76 (20.4%)	111 (20.9%)		3 (13.6%)	9 (32.1%)	
complex cystic and solid	8 (2.2%)	17 (3.2%)		2 (9.1%)	1 (3.6%)	
hypoechoic	288 (77.4%)	402 (75.9%)		17 (77.3%)	18 (64.3%)	
posterior features			<0.001			0.045
enhancement	59 (15.9%)	144 (27.2%)		0	6 (21.4%)	
no posterior features	69 (18.5%)	164 (30.9%)		8 (36.4%)	11 (39.3%)	
shadowing	244 (65.6%)	222 (41.9%)		14 (63.6%)	11 (39.3%)	
calcifications			<0.001			0.004
rim calcification	10 (2.7%)	7 (1.3%)		0	1 (3.6%)	
no calcification	75 (20.2%)	275 (51.9%)		1 (4.5%)	12 (42.9%)	
macrocalcification	66 (17.7%)	231 (43.6%)		8 (36.4%)	10 (35.7%)	
microcalcification	221 (59.4%)	17 (3.2%)		13 (59.1%)	5 (17.8%)	
architectural distortion			<0.001			0.001
no	23 (6.2%)	319 (60.2%)		4 (18.2%)	19 (67.9%)	
yes	349 (93.8%)	211 (39.8%)		18 (81.8%)	9 (32.1%)	
suspicious lymph node			<0.001			0.047
no	110 (29.6%)	487 (91.9%)		6 (27.3%)	16 (57.1%)	
yes	262 (70.4%)	43 (8.1%)		16 (72.7%)	12 (42.9%)	
duct changes			0.032			0.393
no	159 (42.7%)	264 (49.8%)		10 (45.5%)	17 (60.7%)	
yes	213 (57.3%)	266 (50.2%)		12 (54.5%)	11 (39.3%)	
hyperechoic halo			0.855			1.000
no	247 (66.4%)	355 (67.0%)		16 (72.7%)	20 (71.4%)	
yes	125 (33.6%)	175 (33.0%)		6 (27.3%)	8 (28.6%)	

SLN, sentinel lymph node.

### Choosing a machine learning model

The intra-observer K value of radiologist A was 0.887–0.938 in the two evaluations of the ultrasound signs of lesions, and the inter-observer K value of radiologists A and B was 0.876–0.917. This shows that the evaluation of ultrasound signs was stable and reproducible. All results of this study are based on the ultrasound features of the lesion as assessed by radiologist A.

**Figure 2:**
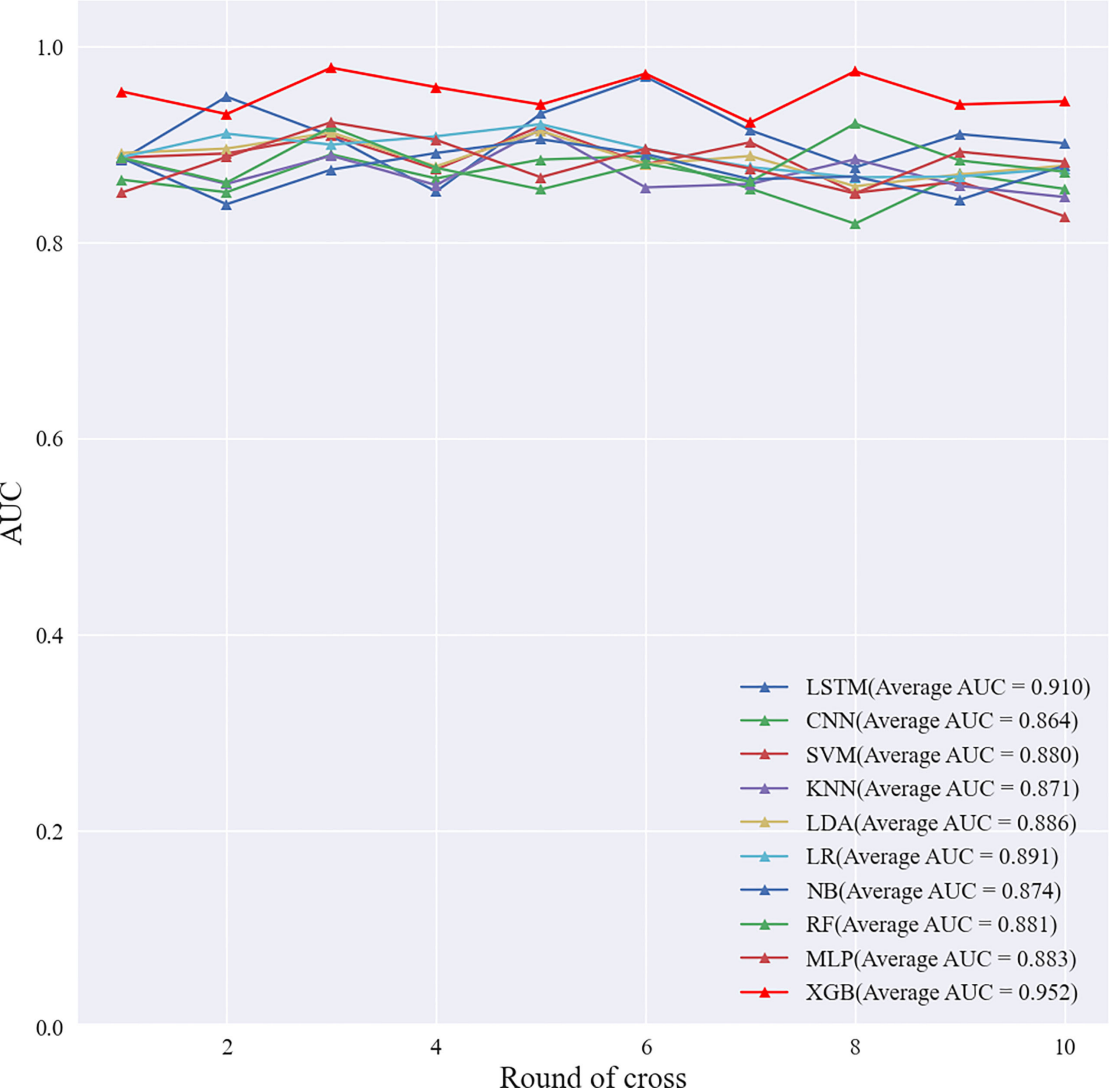
The 10-fold cross-validation of machine learning algorithms.

**Figure 3:**
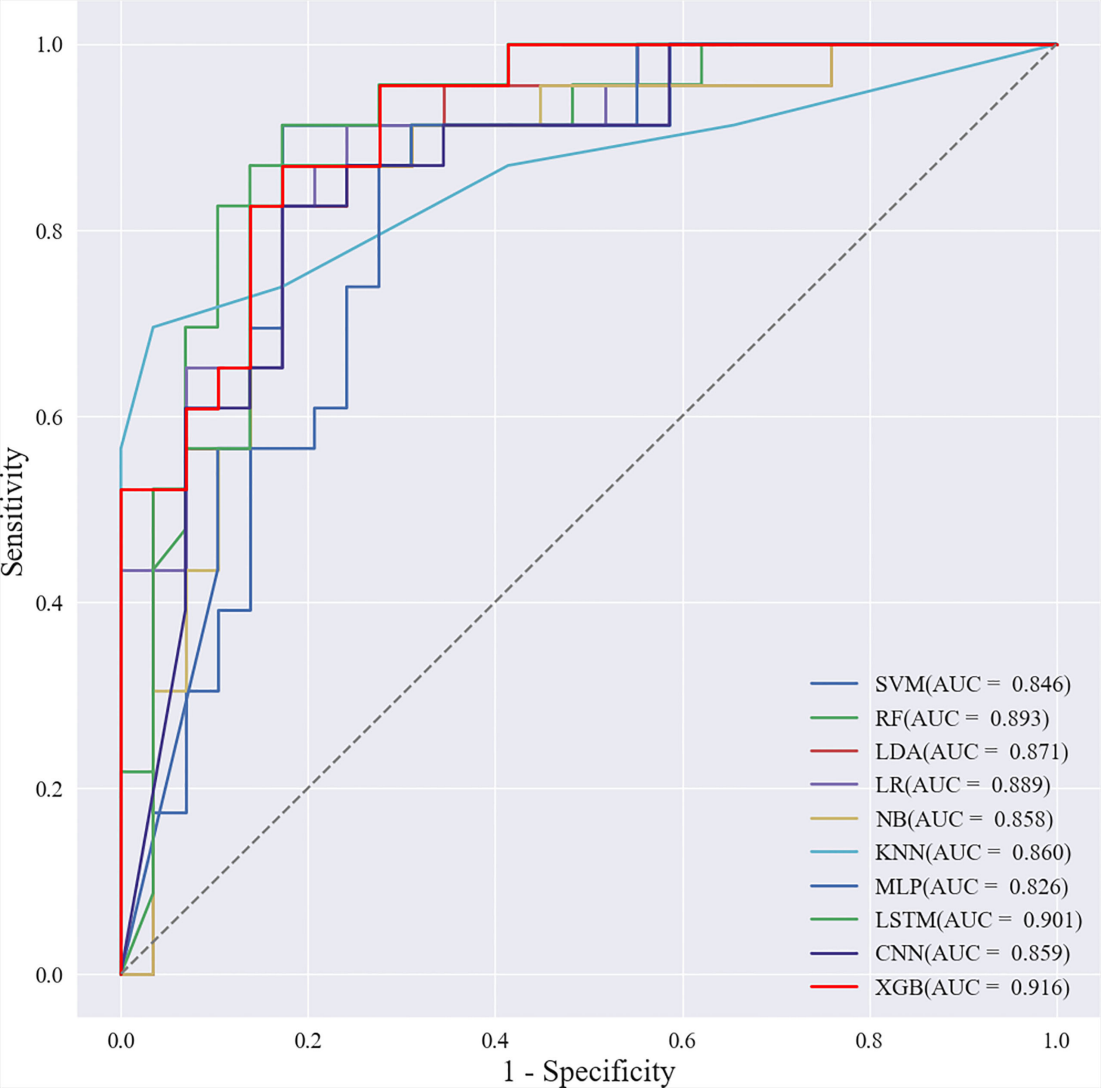
ROC curves of the validation cohort.

**Figure 4:**
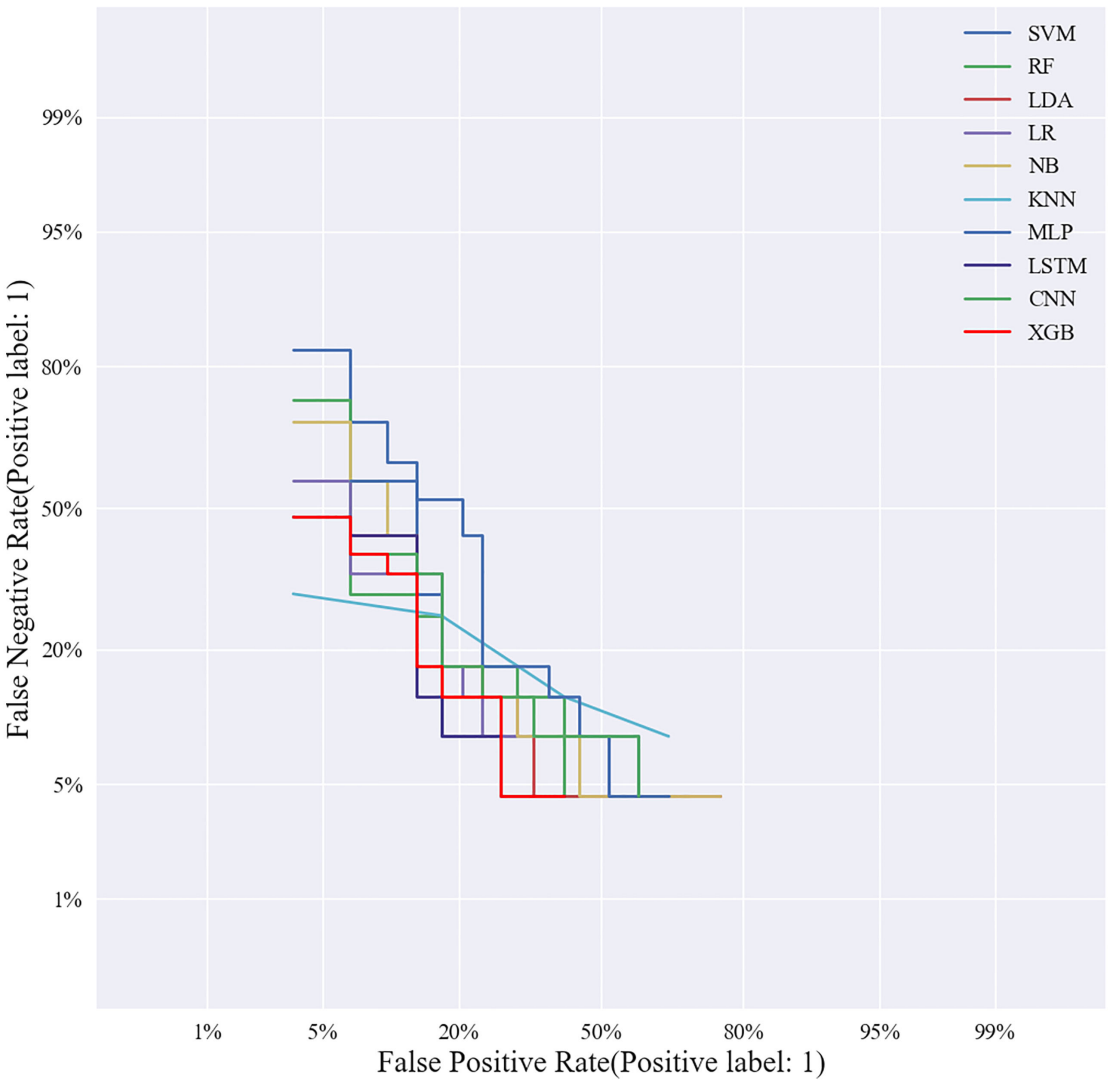
Validation cohort DET curves of 10 machine learning models.

**Figure 5:**
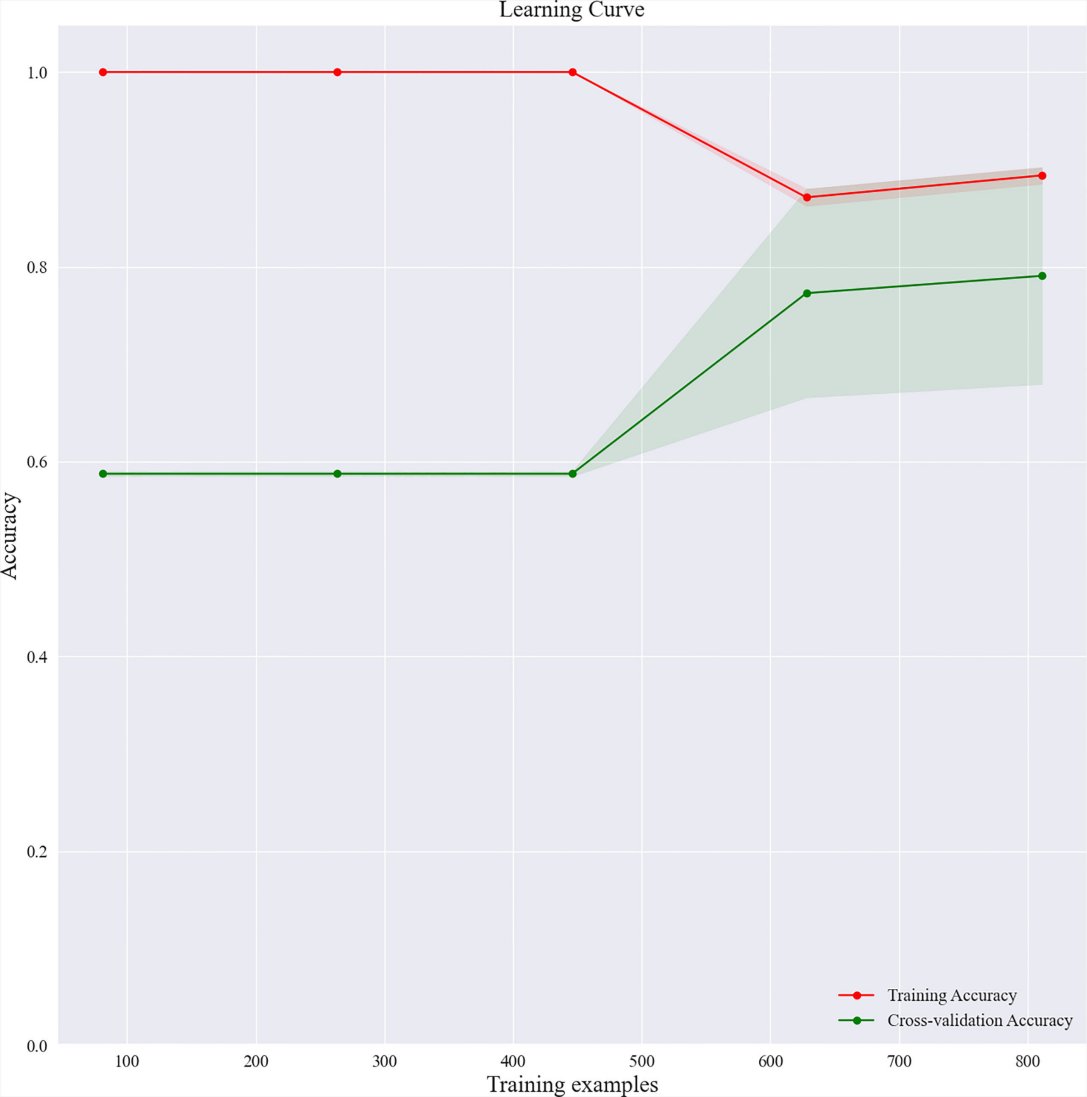
Learning curve of the XGBoost model.

Based on the screened eight key ultrasound signs, the ROC curves of 10 ML models of SVM, XGBoost, RF, LDA, LR, NB, KNN, MLP, LSTM and CNN were compared by 10-fold cross-validation (**Figure 2**). And the models were screened by Average-AUC, Average-Kappa and Average-Accuracy (**Table 3**). We found that the XGBoost model had the best Average-AUC (0.952), Average-Kappa (0.763) and Average-accuracy (0.891). Subsequently, we used the entire data from the primary cohort to train 10 ML models. Finally, external validation of the model used validation cohort has showed that the diagnostic performance of the XGBoost model was still the best (**Figure 3**). The AUC of the model was 0.916, the accuracy was 0.846, the sensitivity was 0.870, the specificity was 0.862, and the F1-score was 0.826 (**Table 4**). This further justifies the correctness of our model selection and experimental procedures. The DET curve shows that the false rejection rate and false acceptance rate of the XGBoost model are lower than other models (**Figure 4**). Additionally, we verified the fit of the model using the learning curve, and the XGBoost model showed a good fit (**Figure 5**). The **Supplementary Material 2** shows the modeling process of the XGBoost algorithm. Finally, we selected the XGBoost algorithm, with the best diagnostic performance, and compared it with the diagnoses from radiologist A.

**Table 3:** Screening Evaluation Metrics for Machine Learning Algorithms Using 10-fold cross-validation.

Classifier	LSTM	CNN	SVM	KNN	LDA	LR	NB	RF	MLP	XGB
Average-AUC	0.910	0.864	0.880	0.871	0.886	0.891	0.874	0.881	0.883	0.952
Average-Kappa	0.717	0.652	0.672	0.656	0.691	0.702	0.624	0.706	0.698	0.763
Average- Accuracy	0.877	0.833	0.794	0.811	0.852	0.823	0.857	0.744	0.779	0.891

AUC, area under curve; SVM, support vector machine; XGBoost, extreme gradient boosting; RF, random forest; LDA, linear discriminant analysis; LR, logistic regression; NB, naive bayesian model; KNN, k-nearest neighbors; MLP, multilayer perceptron; LSTM, long short-term memory; CNN, convolutional neural network.

**Table 4:** Externally validate the performance of machine learning models.

Classifier	AUC	Accuracy	Sensitivity	Specificity	F1-score
LSTM	0.901	0.826	0.957	0.724	0.830
CNN	0.859	0.826	0.826	0.828	0.809
SVM	0.846	0.788	0.739	0.828	0.756
KNN	0.860	0.788	0.739	0.828	0.756
LDA	0.871	0.827	0.780	0.862	0.800
LR	0.889	0.800	0.826	0.759	0.800
NB	0.858	0.846	0.826	0.862	0.826
RF	0.893	0.711	0.913	0.552	0.737
MLP	0.826	0.750	0.696	0.793	0.711
XGB	0.916	0.846	0.870	0.862	0.826

AUC, area under curve; SVM, support vector machine; XGBoost, extreme gradient boosting; RF, random forest; LDA, linear discriminant analysis; LR, logistic regression; NB, naive bayesian model; KNN, k-nearest neighbors; MLP, multilayer perceptron; LSTM, long short-term memory; CNN, convolutional neural network.

### Factors affecting diagnostic performance of XGBoost model

**Figure 6:**
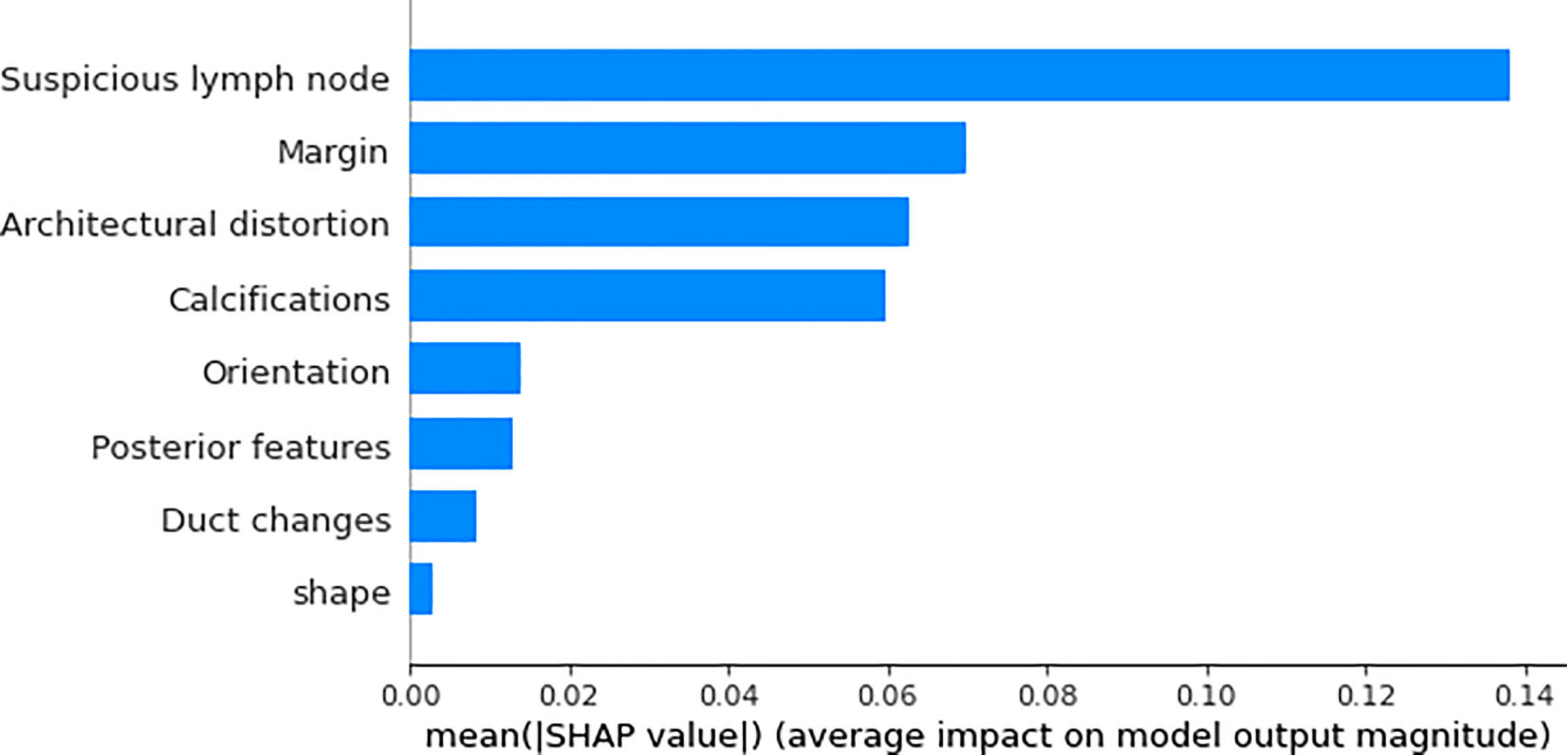
The bar graph of the SHAP summary graph shows the effect of each ultrasound sign on the XGBoost model. “Suspicious lymph node” was the factor that contributed the most to the prediction result, and margin, architectural distortion, and calculations also had a higher contribution to the prediction result.

**Figure 7:**
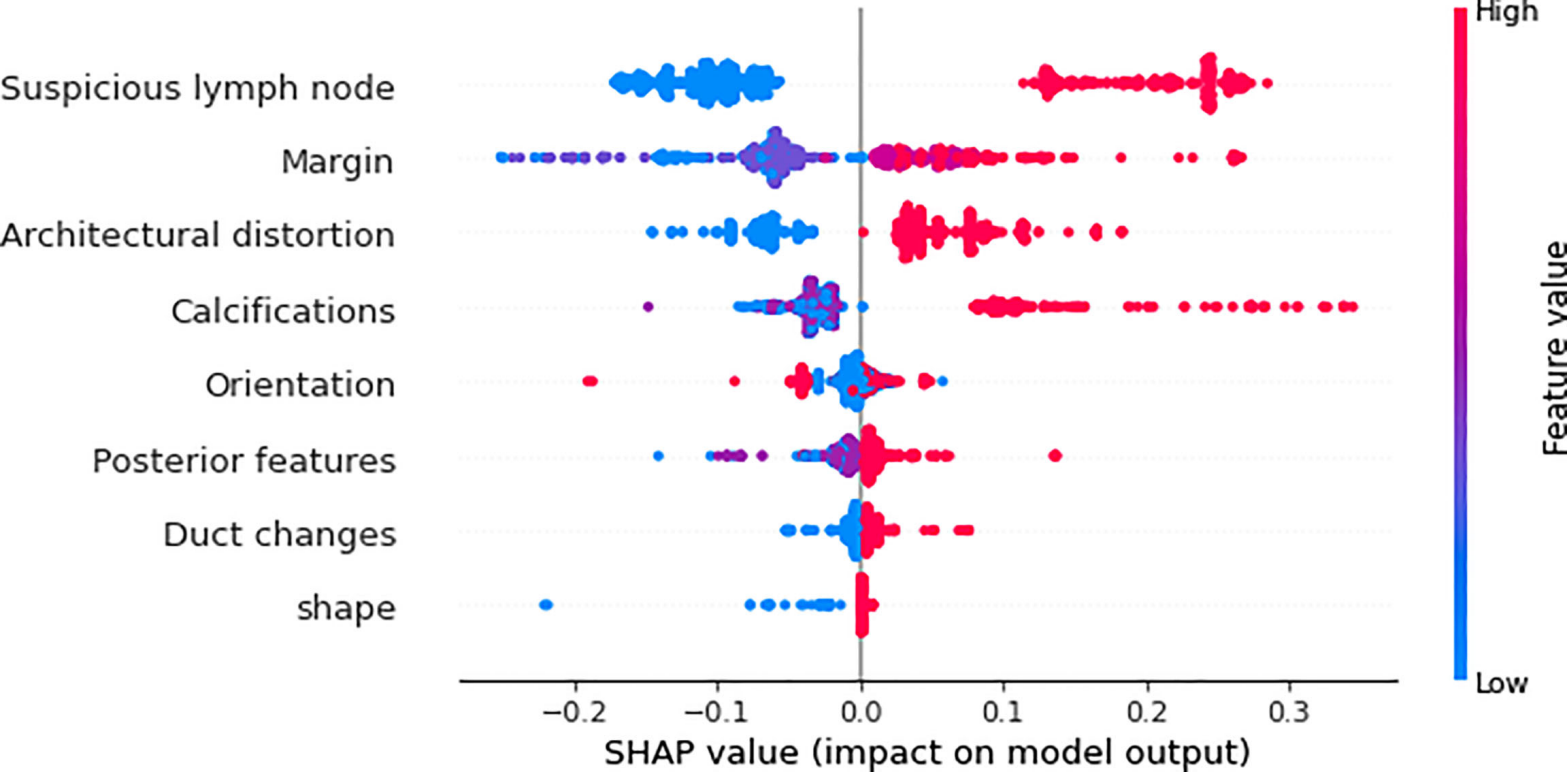
The scatter plot of the SHAP summary chart visually reflects the relationship between the feature value and the predicted probability through color, including positive and negative prediction effects. The three signs of “suspicious lymph node,” “architectural distortion,” and “calculations” are very clearly divided, and the margin is relatively clear. The higher the value (red), the greater the possibility of SLN transfer.

**Figure 8:**
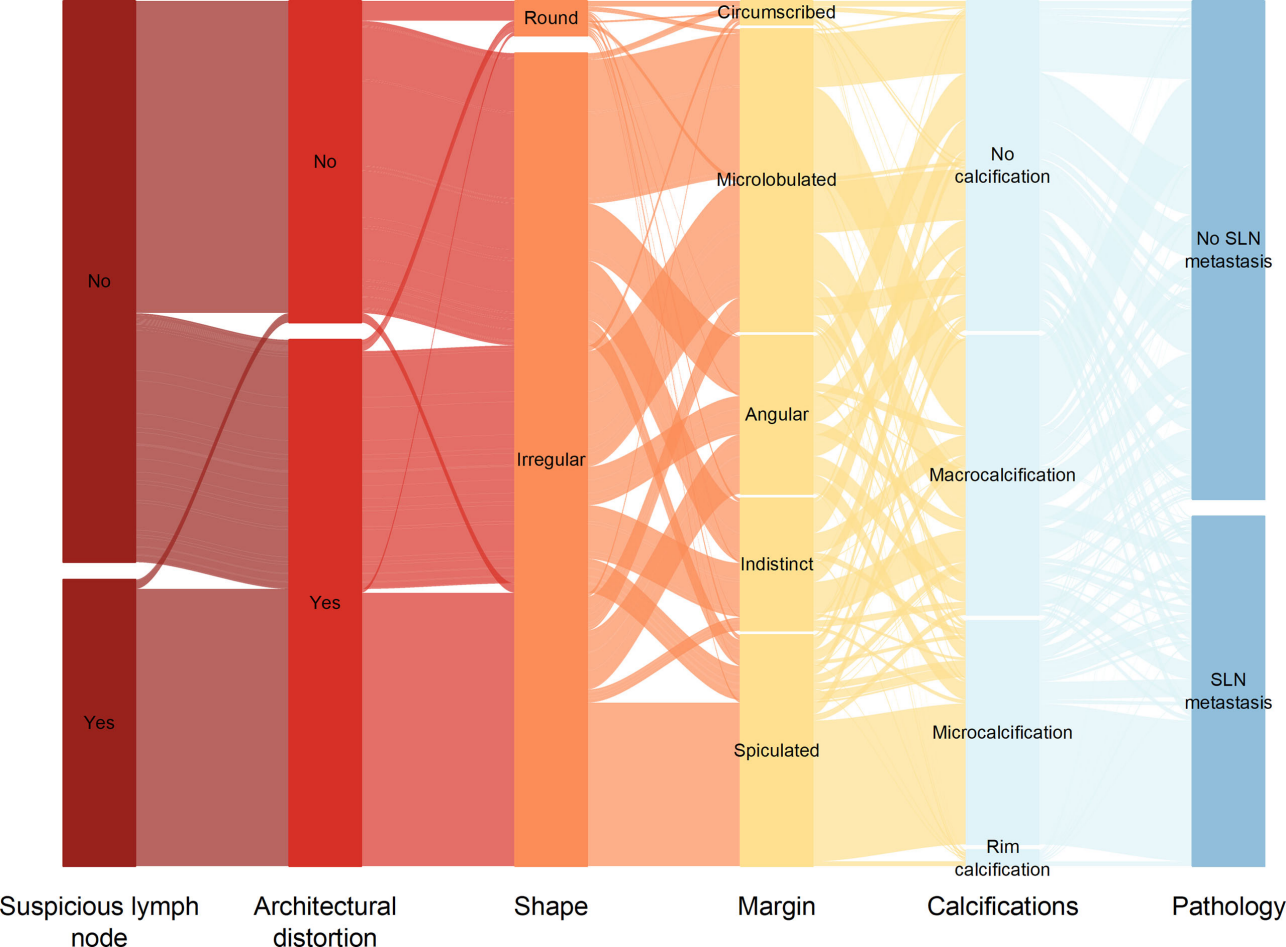
Sankey plot shows the distribution of ultrasound signs of breast cancer lesions in the primary cohort.

**Figure 9:**
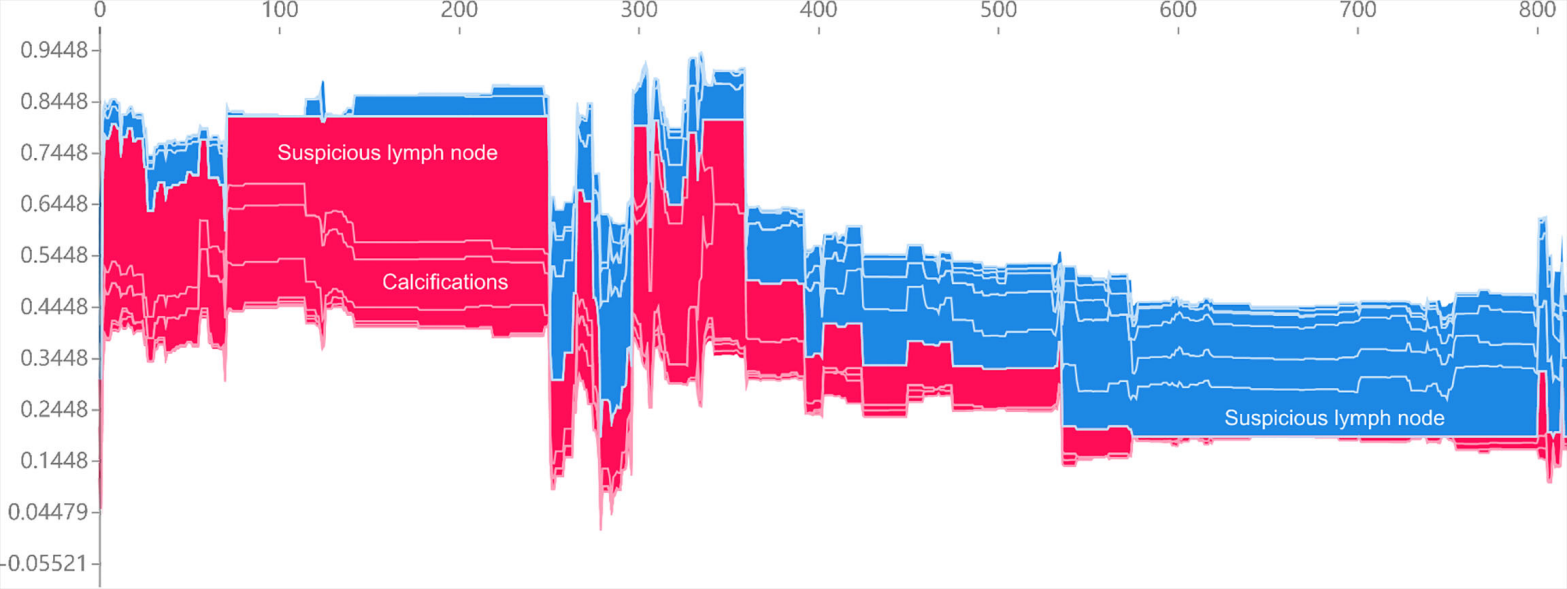
The force plot of the SHAP summary plot reflects the positive or negative impact of the eigenvalues on the diagnosis of the XGBoost model in red and blue.

**Figure 10:**
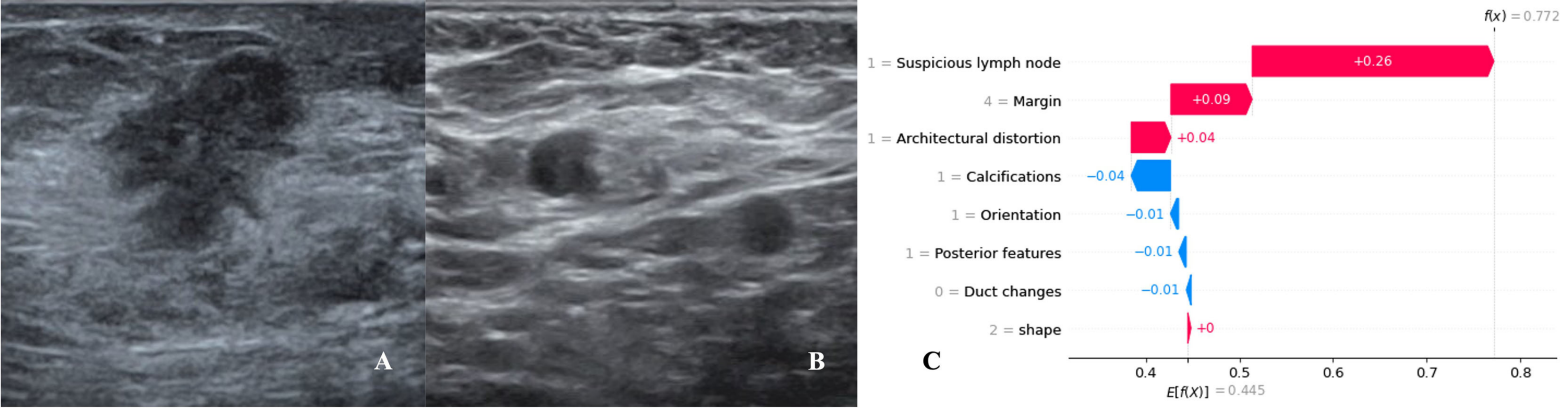
Data from a female patient, 46 years old. **(A)**. Right breast probing and hypoechoic lesions, not parallel to the skin, irregular in shape, burr-like edges, and disordered echoes of surrounding structures; **(B)**. Right axillary probing and echoes of suspicious lymph nodes. Pathological findings: invasive ductal carcinoma, metastases in sentinel lymph nodes; **(C)**. The waterfall chart of the XGBoost model predicted the process of SLN metastasis in this case. For this patient, the predicted outcome was 77.2% (baseline: 44.5%), and high-risk factors for being diagnosed with SLN metastasis included suspicious lymph nodes, spiculated lesion margins, and architectural distortion.

**Figure 11:**
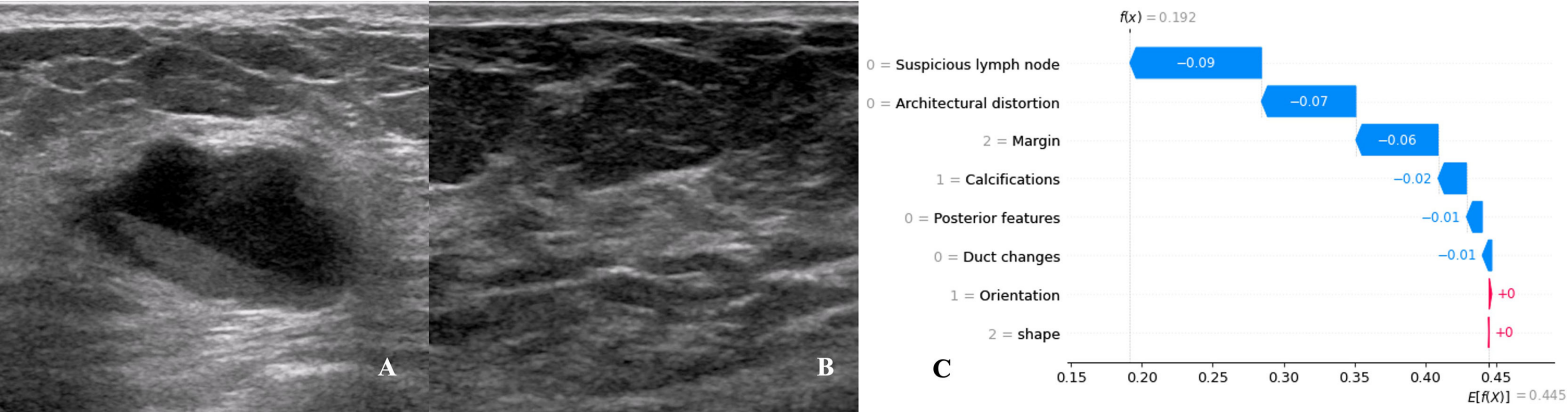
Data from a female patient, 62 years old. **(A)**. Right breast probing and mixed echogenic lesions, not parallel to the skin, irregular in shape, lobulated at the edge, and echogenic in the rear; **(B)**. No suspicious lymph node echo was detected in the right axilla. Pathological findings: invasive ductal carcinoma, no metastases in sentinel lymph nodes; **(C)**. The waterfall chart of the XGBoost model predicting the process of SLN metastasis in this case. For this patient, the predicted outcome was 19.2% (the baseline was 44.5%), and the favorable factors mainly included the margin of the lesion being lobulated, no suspicious lymph nodes being found, no obvious distortion of the tissue structure around the lesion, and no calcification in the lesion.

We utilized SHAP to visualize the XGBoost model results. The SHAP bar graph (**Figure 6**) was obtained by analyzing the mean value of the absolute SHAP values of eight ultrasound signs to show the degree of influence on the final predicted probability. The SHAP scatterplot (**Figure 7**) shows the positive or negative impact of each ultrasound sign on the predicted probability through different colors. We found that suspicious lymph nodes, with microcalcifications, spiculation at the edge of the lesion, and distorted tissue structure around the lesion, had a greater positive impact on the diagnosis of SLN metastasis in the XGBoost model. A Sankey diagram shows the distribution of key ultrasound signs in the primary cohort (**Figure 8**). The SHAP effort plot (**Figure 9**) demonstrates the cumulative effect of the contribution of each ultrasound sign in the primary cohort on the final decision. **Figures 10**, **11** show two examples of correctly predicted SLN transfer and no transfer, respectively. SHAP waterfall plots (**Figures 10C**, **11C**) demonstrate the positive and negative effects of each ultrasound sign on the predicted outcome in a single case. E[f(x)] represents the basic prediction probability of the XGBoost model, and f(x) represents the final prediction probability of the model.

### Comparison of diagnostic performance of XGBoost model with radiologists

**Figure 12:**
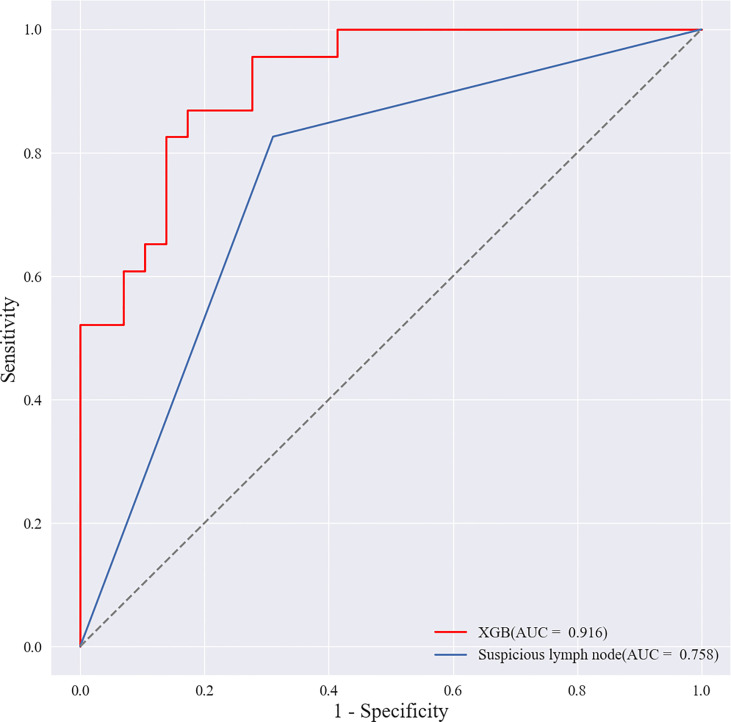
Receiver operating characteristic (ROC) curves of XGBoost models and radiologists. The areas under the curve (AUCs) of the two methods (0.916 vs. 0.758) were significantly different as determined by the DeLong method (P<0.001).

Radiologist A considered suspicious lymph nodes detected by ultrasound as the presence of SLN metastasis based on the lymph node ultrasound appearance and clinical experience, and we compared the diagnostic performance of the radiologists and the XGBoost model in the validation cohort. It was found that the AUC of the XGBoost model was 0.916, while the AUC of the radiologist was 0.758 (**Figure 12**). The difference was significant as determined by the DeLong method (P<0.001).

## Discussion

This study retrospectively analyzed the ultrasound signs and pathological findings of a total of 952 breast cancer lesions in primary and validation cohorts. Using these data to screen ten common ML algorithms, it was found that the comprehensive diagnostic performance of the XGBoost model was the best and was higher than that of experienced radiologists (P<0.001). Suspicious lymph nodes, microcalcifications, spiculation signs at the edge of the lesion, and structural distortion around the lesion had a greater impact on the diagnostic performance of the XGBoost model and are the key ultrasound signs for predicting SLN status. We further used SHAP to reasonably explain the prediction results of the XGBoost model, which provides a reliable auxiliary tool for clinical decision-making.

Previous studies have mostly used logistic regression to construct nomogram clinical prediction models by extracting ultrasound image features (17). Logistic models have good interpretability, and their model coefficients represent the importance of features to prediction results. A study (18) compared the predictive ability of classification trees, random forests, artificial neural networks, and support vector machines in the ML algorithm with logistic regression and found that the predictive ability of logistic regression was equally excellent. However, this may not be statistically significant due to some factors that have a causal relationship to the output variable (19). Excluding variables based solely on statistical assumptions reduces available information and may miss features that improve predictive power. In addition, the logistic regression model has low accuracy and is limited in practical clinical application (20). This study found that the Average-AUC (0.952), Average-Kappa (0.763) and Average-Accuracy (0.891) of the XGBoost model in the 10-fold cross-validation were higher than those of Logistic regression. The XGBoost model in the validation cohort also performed well, with AUC of 0.916, accuracy of 0.846, sensitivity of 0.870, specificity of 0.862, and F1-score of 0.826. We also compared eight other ML algorithms (SVM, RF, LDA, NB, KNN, MLP, LSTM and CNN). The comprehensive diagnostic performance of the XGBoost model was still the best. Therefore, we chose to use the XGBoost algorithm to build a clinical prediction model to achieve the best diagnostic level. Next, we utilized SHAP to solve the interpretability problem of the XGBoost model. Compared with traditional ML model interpretation methods, SHAP not only considers the influence of a single variable but also considers the synergy between different variables and distinguishes the positive or negative influence of variables by color (21).

In this study, SHAP was used to find that suspicious lymph nodes detected by ultrasound had a great impact on the diagnostic performance of the XGBoost model. In addition, ultrasonographic signs such as microcalcification in the lesion, burr-like edges of the lesion, and disordered and distorted tissue structure around the lesion had a significant positive effect on the diagnosis of SLN metastasis by the XGBoost model. This may be because tumor cells infiltrate the surrounding tissues, invading the Cooper’s ligament and the lymph nodes through the lymphatic vessels (22). In this study, suspicious lymph nodes were assigned the largest contribution value in the SHAP map, which is consistent with previous studies (23) in which the detection of suspicious lymph nodes by ultrasound improved the diagnostic specificity of breast cancer SLN metastases. At the same time, Drukker et al. (24, 25) also confirmed that the analysis of ultrasound images of axillary lymph nodes can effectively predict breast cancer metastasis, but the AUCs were 0.85 and 0.86, which were lower than our study (AUC=0.916). It should be considered, however, that this may be because we also included the ultrasound signs of the primary lesions of breast cancer patients to train the ML model, which further increased the diagnostic performance of the model. Li et al. (26) found that breast cancer with calcification had a higher rate of lymph node metastasis. Luo et al. (27, 28) found that the tumor pathological type, tumor burr sign, and calcification characteristics were related to axillary lymph node metastasis. These studies are consistent with the findings of the present study. The tumor edge spiculation sign is often the manifestation of the infiltration and growth of the lesion to the surrounding tissue, suggesting that the tumor is malignant, and its OR value is 14.68-10.45 (29). Compared with coarse calcification, micro calcification is usually one of the indicators of rapid proliferation of cancer cells, and it is also a manifestation of high tumor malignancy (30), which increases the risk of SLN metastasis in breast cancer. In this study, the SHAP map also found that the contribution of architectural distortion to predicting SLN metastasis was second only to suspicious lymph nodes. Architectural distortion usually includes twisting of the ducts around the mass, shortening and straightening of Cooper’s ligament, and the mass breaching the anatomical plane to invade adipose tissue (31). Paulinelli et al. (32) found that Cooper’s ligament thickening is a characteristic of malignant tumors, and its odds ratio value was 15.61. The studies of Woo (33) and Lee (34) also confirmed that the combination of ultrasound images of primary tumor and peritumoral tissue can more effectively predict the status of axillary lymph nodes. We believe that training the XGBoost model with the best diagnostic performance by synthesizing the ultrasound signs of breast cancer lesions, peritumoral tissues, and suspicious lymph nodes is the key to improving the accuracy of SLN metastasis prediction. At the same time, SHAP provides a personalized and reasonable explanation for prediction, breaking the “black-box” problem that has been hindering the development of complex models and significantly improving the application value of clinical models and the confidence of clinicians in the prediction model.

This study also has certain limitations. First, it was a single-center retrospective study, with limited sample size and regionality. Some cases were eliminated due to the quality of lesion images, thus reducing the sample size. Second, the pathological types of breast cancer included in the samples are not comprehensive, which may affect the results of the study. Third, a more detailed classification of the ultrasound signs of the lesion is also required. In the future, this study also needs to incorporate the relevant features of radiomics, and further analyze and study other ML algorithms involved in medicine.

In conclusion, this study more comprehensively incorporates the ultrasound signs of the primary breast cancer and its surrounding soft tissues and lymph nodes, and establishes an XGBoost model to predict the metastasis of SLN and used SHAP to solve the “black-box” problem that hinders the clinical application of ML algorithms. It provides clinicians with a non-invasive, efficient, and convenient method, assists clinicians to understand the state of SLN before surgery, and guides the selection and treatment of surgical methods.

## Data Availability Statement

The raw data supporting the conclusions of this article will be made available by the authors, without undue reservation.

## Author Contributions

GZ, YS, FL participated in the conception and designed this study. ZZ provided administrative support. GZ, PY, YF, XL participated in the collection and arrangement of research data. GZ, QZ completed the analysis and interpretation of the data. GZ wrote the manuscript. All authors contributed to this article and approved the submitted version.

## Funding

This research was supported by the National Natural Science Foundation of China (No.81471809; No.81971639).

## Conflict of Interest

The authors declare that the research was conducted in the absence of any commercial or financial relationships that could be construed as a potential conflict of interest.

## Publisher’s Note

All claims expressed in this article are solely those of the authors and do not necessarily represent those of their affiliated organizations, or those of the publisher, the editors and the reviewers. Any product that may be evaluated in this article, or claim that may be made by its manufacturer, is not guaranteed or endorsed by the publisher.
