# Supplementary material for: A machine learning model based on ultrasound image features to assess the risk of sentinel lymph node metastasis in breast cancer patients: Applications of scikit-learn and SHAP
Source: Front Oncol. 2022 Jul 25;12:944569. doi: 10.3389/fonc.2022.944569 (PMC9359803; doi:10.3389/fonc.2022.944569)
Supplement: Supplementary file 1 [file DataSheet_1.docx]

Supplementary Material

# Data Preprocessing

Feature scaling is a method used to normalize the range of independent variables or features of data. In data processing, it is also known as data normalization and is generally performed during the data preprocessing step. Since the range of values of raw data varies widely, in some machine learning algorithms, objective functions will not work properly without normalization. For example, many classifiers calculate the distance between two points by the Euclidean distance. If one of the features has a broad range of values, the distance will be governed by this particular feature. Therefore, the range of all features should be normalized so that each feature contributes approximately proportionately to the final distance. Another reason why feature scaling is applied is that gradient descent converges much faster with feature scaling than without it. It's also important to apply feature scaling if regularization is used as part of the loss function (so that coefficients are penalized appropriately).

In this study, Scaling to unit length was used to normalize the dataset. Another option that is widely used in machine-learning is to scale the components of a feature vector such that the complete vector has length one. This usually means dividing each component by the Euclidean length of the vector:

$$x^{'}=\frac{x}{\left\| x \right\|}$$

In some applications it can be more practical to use the L1 norm of the feature vector. Our study used L2 norm.

**Standardization (Z-score Normalization)**

In machine learning, we can handle various types of data, e.g., audio signals and pixel values for image data, and this data can include multiple dimensions. Feature standardization makes the values of each feature in the data have zero-mean (when subtracting the mean in the numerator) and unit-variance. This method is widely used for normalization in many machine learning algorithms (e.g., support vector machines, logistic regression, and artificial neural networks). The general method of calculation is to determine the distribution mean and standard deviation for each feature. Next, we subtract the mean from each feature. Then we divide the values (mean is already subtracted) of each feature by its standard deviation.

$$x^{'}=\frac{x-\overline{x}}{\sigma}$$

Where $x$is the original feature vector, $\overline{x}$ is the mean of that feature vector, and $\sigma$is its standard deviation. We applied this method to normalize the dataset.

For the original dataset of this study, please refer to https://pan.baidu.com/s/1ipRB0H6EWkZ_VAVCD0LGhw?pwd=2e94.

# Detailed modeling process of XGBoost algorithm

XGBoost (eXtreme Gradient Boosting) is an algorithm toolkit based on the Boosting framework, which is very superior in parallel computing, missing value processing, and prediction performance. It is an improved and upgraded version of the gradient boosting decision tree algorithm. On its basis, the efficiency and methods have been improved. The basic idea of the algorithm is to grow a tree by continuously adding trees and feature splitting and to use each newly added tree to fit the predicted residual. When the training is completed to predict the sample, it will fall on a corresponding leaf node in each tree according to the characteristics of the sample, and each leaf node corresponds to a score, and finally the scores corresponding to each tree are summed, which is the predicted value for that sample.

This study uses grid search for parameter tuning of various ML algorithms. XGBoost performs the best with learning_rate= 0.0086, max_depth= 5, min_child_weight= 5, n_estimators= 217, subsample= 0.5, and outperforms other ML algorithms after parameter tuning. the XGBoost algorithm with the best diagnostic performance in this sample set was used to predict the metastasis status of sentinel lymph nodes in breast cancer patients. At test time, a sample input feature vector $X_{i}$= ($X_{1}$, $X_{2}$, ……, $X_{n}$) is given, where the features included shape, margin, calculations, etc. In the training process, it is necessary to add trees on the basis of the previous stage. After adding trees, the residual error of prediction will be reduced, and the prediction performance will be improved. The following equations show the training process of XGBoost.

The XGBoost algorithm introduces a regularization penalty term to prevent overfitting. Finally, the objective function of XGBoost consists of its own loss function and regularization penalty term, as follows:

$$obj=\sum_{i} l(y_{i}, \hat{y}_{i})+\sum_{t} \Omega(f_{t})$$

Among them，$l(y_{i}, \hat{y}_{i})$represents its own loss function, and$\Omega$ represents the regularization penalty term, specifically:

$$\Omega\left( f_{t} \right)= \gamma T+\frac{1}{2}\sum_{j=1}^{T} \omega_{j}^{2}$$

Among them, T represents the number of leaves; $\omega_{j}$ represents the weight of the leaf nodes; the variable $\gamma$is the punishment intensity, which can control the score of the leaf nodes not to be too large and prevent overfitting; is a constant that controls the degree of regularization.

XGBoost supports linear classifiers, namely logistic regression and linear regression equivalent to L1 and L2 regularization terms. For the operation of the second-order Taylor expansion of the cost function, the first-order derivative and the second-order derivative are introduced. When the sample has missing values, it can automatically learn the split direction. At the same time, the random forest method is used to support column sampling, which can prevent overfitting and reduce computational complexity. The cost function introduces a regularization term, which reduces the complexity of the model. The regularization term includes the number of all leaf nodes. From the perspective of Bayesian variance, the regular term reduces the variance of the model and prevents the model from overfitting. XGBoost supports parallelism to improve computing efficiency.
